# 4DCT and VMAT for lung patients with irregular breathing

**DOI:** 10.1002/acm2.13453

**Published:** 2021-11-24

**Authors:** Rhydian Caines, Naomi K. Sisson, Carl G. Rowbottom

**Affiliations:** ^1^ Medical Physics Department Clatterbridge Cancer Centre Liverpool UK

**Keywords:** 4DCT, breathing, irregular, lung, radiotherapy, VMAT

## Abstract

**Purpose:**

Irregular breathing in lung cancer patients is a common contra‐indication to 4D computerized tomography (4DCT), which may then limit radiotherapy treatment options. For irregular breathers, we investigated whether 3DCT or 4DCT (1) better represents tumor motion, (2) better represents average tumor densities, and (3) better allows for volumetric modulated arc threarpy (VMAT) plans delivered with acceptable dosimetric accuracy.

**Methods:**

Ten clinical breathing traces were identified with irregularities in phase and amplitude, and fed to a programmable moving platform incorporating an anthropomorphic lung tumor phantom. 3DCT and 4DCT data resorted by phase (4DCT‐P) and amplitude (4DCT‐A) were acquired for each trace. Tumors were delineated by Hounsfield unit (HU) thresholding and apparent motion range assessed. HU profiles were extracted from each image and agreement with calculated expected profiles quantified using area‐under‐curve (AUC) scoring. Clinically representative VMAT plans were created for each image, delivered to the irregularly moving phantom, and measured with a small‐volume ion chamber at the tumor center.

**Results:**

Median difference from expected tumor motion range for 3DCT, 4DCT‐P, and 4DCT‐A was 2.5 [1.6–3.6] cm, 1.1 [0.1–1.9] cm, and 1.3 [0.4–1.9] cm, respectively (*p* = 0.005, 4DCT‐P vs. 3DCT). Median AUC scores (ideal = 0) for 3DCT, 4DCT‐P, and 4DCT‐A were 0.25 [0.14–0.49], 0.12 [0.05–0.42], and 0.13 [0.09–0.44], respectively (*p* = 0.005, 4DCT‐P vs. 3DCT). Nine of ten 4DCT‐P plans and all 4DCT‐A plans measured within 2.5% of expected dose in the treatment planning system (TPS), compared with seven 3DCT plans.

**Conclusion:**

For the cases studied tumor motion range and average density was better represented with 4DCT compared with 3DCT, even in the presence of irregular breathing. 4DCT images allowed for delivery of VMAT plans with acceptable dosimetric accuracy. No significant differences were detected between phase and amplitude resorting. In combination with 4D cone beam imaging at treatment, our findings have given us confidence to introduce 4DCT and VMAT for lung radiotherapy patients with irregular breathing.

## 1 INTRODUCTION

4DCT is an established technique for simulating internal respiratory motion for lung cancer patients undergoing radiotherapy.^1^, ^2^, ^3^, ^4^, ^5^ Typically, a low‐pitch CT acquisition is undertaken simultaneously with the recording of an external breathing surrogate signal, such as a reflective block placed on the abdomen. Images acquired over multiple breathing cycles are retrospectively sorted during reconstruction into “bins” of equal phase or equal amplitude and a time‐dependent volumetric image created. By explicitly imaging the tumor motion envelope, residual setup uncertainties are reduced permitting reduction in planning target volume (PTV) margin and potentially the volume of normal tissue that is irradiated.^6^ In this way, the benefits of modern, highly conformal RT techniques such as VMAT are enhanced.

The most common source of artefact in 4DCT is irregular breathing.^7^ In such cases, the assumptions underlying commercially available 4D reconstruction algorithms are violated, since a given phase may contain a large range of amplitudes, or vice versa, resulting in inaccurate or insufficient representations of the tumor motion. Unfortunately, irregular breathing is not uncommon among lung cancer patients owing to clinical factors and comorbidities typical in this patient cohort.^8^, ^9^ In addition breathing irregularity may be induced during the scanning session due to anxiety and other physiological factors.^10^, ^11^

The existing literature is divided on the best way to manage breathing irregularity in 4DCT. Mainly, caution is advised owing to the potential to either under‐ or over‐estimate the range of tumor motion in the patient.^12^, ^13^ Some authors have advocated devising individualized patient PTV margins to compensate for this.^14^, ^15^ This of course carries a resource implication and potentially a clinical risk associated with non‐standardized practice. A so‐called 5DCT technique has been proposed that combines a recorded surrogate signal with multiple high‐pitch 3DCT scans and deformable image registration maps parameterized by breathing amplitude and breathing rate.^16^, ^17^ This approach has recently been shown to produce artefact‐free images for irregular breathers,^18^ though is not yet commercially widely available.

While there has been much discussion regarding accuracy and artifacts of 4DCT images acquired during irregular breathing,^7^, ^12^, ^14^, ^19^ few studies have evaluated their dosimetric quality—in particular their suitability or otherwise for treatment planning and treatment delivery. This leaves radiotherapy clinics with a quandary when developing scanning protocols: when the patient is breathing irregularly, what is the most appropriate imaging modality and management approach? Local implementation of existing guidance has historically been to 3DCT patients that breathe irregularly (if they could not be otherwise coached), then increase PTV margins to approximately compensate for the additional uncertainty. This would potentially increase the volume of the normal tissue in the treatment field, and in some cases limit the prescribed dose.

The present work therefore addresses the question of how best to simulate a lung cancer patient for treatment planning in the presence of irregular breathing. In particular given the clinically available options of 3DCT, and 4DCT with resorting by either phase (4DCT‐P) or amplitude (4DCT‐A), we investigated which modality (a) best represents the range of tumor motion, (b) best represents the average densities in the patient, and (c) yields the most appropriate images for accurate dose planning, when the patient is unable to breathe regularly.

## 2 METHODS

### 2.1 3D and 4D image creation

#### 2.1.1 Breathing trace selection

Our local 4D scanning protocols use Varian's respiratory gated scanning (RGSC) software for respiratory monitoring, assessment, and processing of 4DCT data (Varian Medical Systems, Palo Alto, CA). The patient's breathing trace is monitored before and during scanning with a reflective marker block placed on the abdomen. This software has an integrated online respiratory predictive filter which together with clinical experience of the pre‐treatment radiographers can be used to determine if breathing is regular. The predictive filter sensitivity can be adjusted with 100% requiring perfect reproducibility and 0% corresponding to the filter being off. If the trace is reproducible at a specified sensitivity level, the trace is rendered in real time as a black line, turning red when irregularities occur. Varian recommends a predictive filter setting of 5% for 4D reconstruction. Locally, we use 5% as standard; the pre‐treatment radiographers assess the rendered breathing trace and if there are significant regions of red, the predictive filter is lowered to 2%. If there are still significant regions of red coaching would be attempted for these patients. For those patients unable to maintain sufficiently regular breathing even under coaching, our local protocols specify a 3DCT which is then acquired instead of a 4DCT.

**FIGURE 1:**
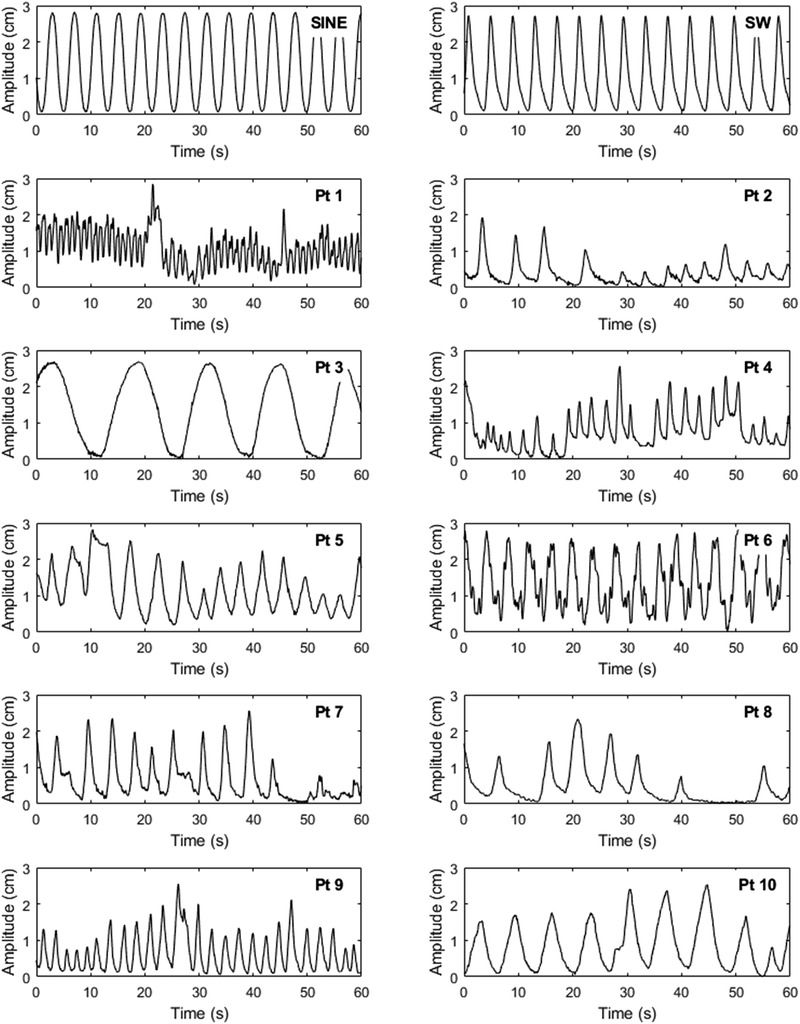
The sine, single wave, and clinical irregular breathing traces used for the study illustrating the first 60 s of each trace

We felt that it was essential to our study to use real patient breathing traces with real irregularities rather than simulated traces. Therefore, 10 breathing traces were selected for inclusion retrospectively, from patients who had irregular breathing when assessed under the above protocol (Pt 1–Pt 10, Figure 1). Therefore, none of these patients were originally eligible for 4DCT without coaching. Of these patients, three were able to achieve 4DCT with audio coaching, while the remaining seven did not tolerate coaching and received a 3DCT. Two further breathing traces were used in this study for reference and calibration of the analysis techniques; the first was a pure sine wave (“SINE”) and the second was a single human breathing trace played “on repeat” (“SW”) so that the breathing was both regular and clinically representative (Figure 1).

#### 2.1.2 Phantom

**FIGURE 2:**
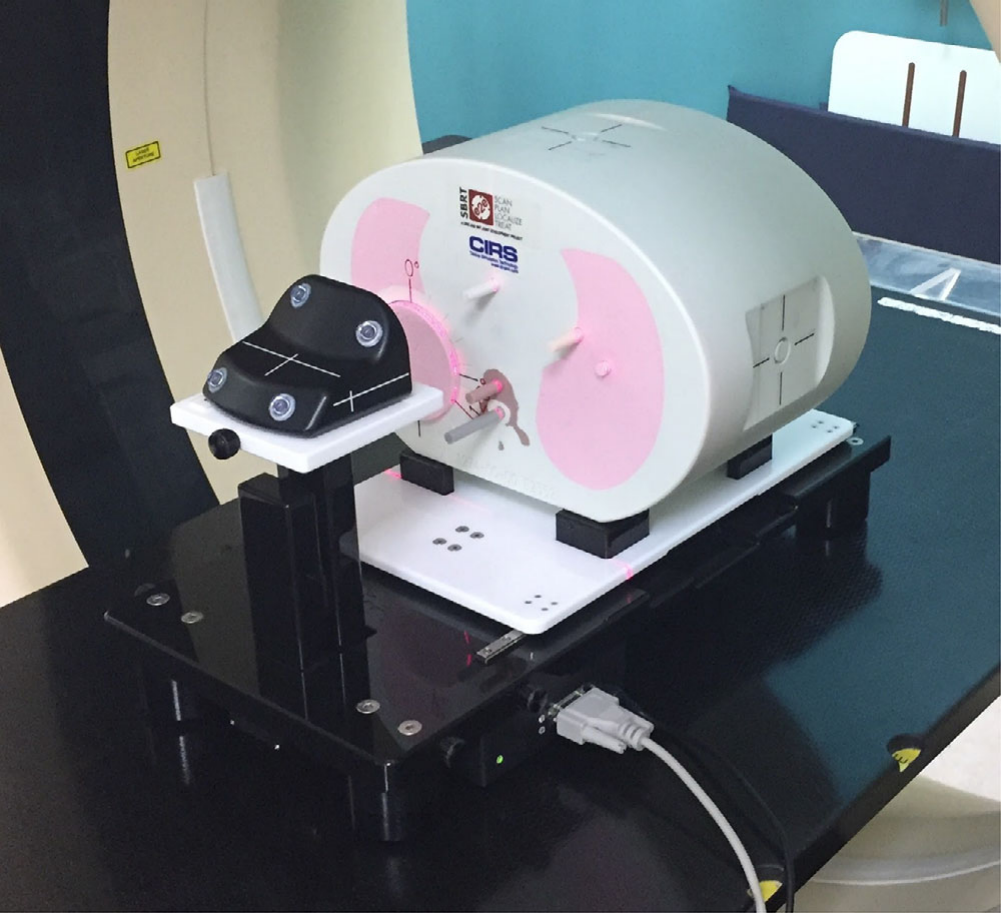
Photograph of BrainLab ET Gating Phantom with CIRS lung phantom set up on the CT scanner couch

The study phantom (Figure 2) was comprised of two parts; a programmable Brainlab ET Gating Phantom (Brainlab, Munich, Germany) and a CIRS anthropomorphic lung phantom (CIRS, Norfolk, Virginia). The Brainlab phantom simulates patient respiratory motion using two synchronized moving platforms. The first platform moves horizontally simulating internal respiratory motion, i.e., the diaphragm and tumor moving in the sup–inf direction. This platform has a range of travel of up to 2.85 cm. The second platform moves vertically to simulate external respiratory motion, i.e., the chest expanding and contracting. The CIRS anthropomorphic phantom was placed on the first platform and the Varian RGSC reflective marker block placed on the second. The CIRS lung phantom is a bespoke anthropomorphic phantom designed for our center. It contains lung, bone, and tissue equivalent materials to represent the thorax, with a spherical tumor within each lung. The tumors are 3 cm and 2 cm in diameter. The 2 cm tumor also incorporates an insert that can be replaced by a PTW small volume (0.015 cm^3^) “PinPoint” ionization chamber (PTW, Freiburg, Germany), permitting dosimetric measurements at the tumor center during treatment delivery. The resulting composite phantom—combining size of tumor with motion range—is representative of the more extreme cases of motion that we encounter clinically. Each of the selected breathing traces could then be fed to the Brainlab phantom via the included control software. When sending a programmed breathing trace back to the phantom both platforms moved, in phase with each other, simulating the correlated motion of the two primary components (ant–post and sup–inf) seen in clinical cases. Although the clinical breathing traces are in fact 3D composite signals of three spatial components of motion, decomposition back into these components was not possible retrospectively. Therefore, while the expected primary components of motion typically seen clinically were captured, our phantom represents a simplified model of human respiratory motion. In each case, the breathing trace amplitudes were scaled by the software such that when evaluated over the whole breathing trace the peak inspiration and expiration separation corresponded with the maximum range of travel on the moving platform. This was verified by measurement prior to imaging. The “expected” breathing amplitude for comparison was set to the middle 95% of the total distribution of the time‐series of amplitudes to avoid extreme outlier breaths such as coughs (on average 2.14 ± 0.36 cm across the cohort).

#### 2.1.3 3D and 4D acquisition

CT imaging was performed using a Philips Brilliance Big Bore CT (Philips, Amsterdam, The Netherlands) with the RGSC respiratory monitoring system. The moving phantom was positioned in the scanner and images acquired using our local clinical 3D and 4D imaging protocols. For all the scans, slice collimation in the axial direction was 16 × 1.5 mm. The 3D scans used a rotation time of 0.75 s and pitch of 0.813. For the 4D scans, rotation time was set to 0.44s, and pitch values selected according to nominal detected breathing rate from the RGSC system, a range of 0.04–0.09 was used in this study. The 4D data were resorted into six bins equally spaced in (1) phase (4DCT‐P) and (2) amplitude (4DCT‐A). Therefore, 36 volumetric images were generated in total (12 breathing traces × 3 scan types). As per the clinical process, all scans were acquired at effectively random points during any given breathing trace, that is, no attempt was made to identify or sample *particularly* irregular subsections of the whole trace, although retrospectively the portions of each breathing trace relevant to each scan were identified and recorded. No gating techniques were used throughout this study. Finally, each 4D image was used to create maximum intensity projections (MIPs) and average intensity projections (AIPs).

### 2.2 Image analysis

The final images were analyzed to assess how well they represented the relevant breathing traces used in terms of both motion range and time‐averaged density.

#### 2.2.1 Tumor motion range measurement

The 3 cm tumor was identified in each image and gross tumour volume (GTV) or internal GTV (iGTV) delineated on the 3D images and 4D‐MIPs respectively using HU thresholding with manual review to confirm the absence of artifacts. The apparent tumor motion range was determined from the sup–inf extent of the GTV, accounting for the known tumor diameter. This was compared to an “expected” range for the scanning session derived from the corresponding breathing trace as the 95th percentile of the total motion evaluated over the whole breathing trace. In this way, outlier tumor positions due to atypical breaths (such as coughing) were excluded.

#### 2.2.2 Tumor density representation

**FIGURE 3:**
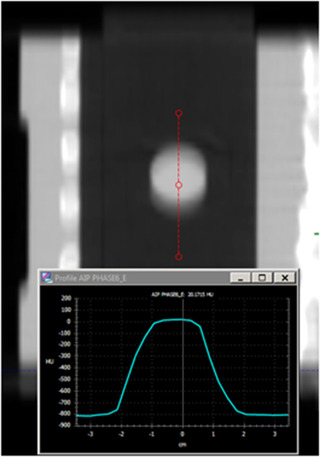
4DCT‐P AIP image showing tumor density profile extracted using Eclipse (Pt 9)

For each image, an HU line profile through the middle of the tumor in the direction of motion was extracted in the Eclipse TPS (Varian Medical Systems, Palo Alto, CA; Figure 3). Expected time‐averaged density profiles were obtained by converting each breathing trace to an amplitude probability density histogram and convolving the resulting function with a density profile though the tumor obtained from a 3DCT scan of the phantom without motion. The expected and measured HU profiles were registered using a mean gamma score^20^ minimization routine (20%, 2 mm) in MATLAB (MathWorks, Natick, MA). Finally, a numerical quality of fit score was obtained by calculating the residual area under the curve normalized to the integral of the expected HU profile (ideal = 0).

### 2.3 VMAT treatment planning and dosimetry

#### 2.3.1 Treatment planning

To determine dosimetric suitability of each image, treatment plans were created according to our local radical lung protocol. GTV/iGTV structures were delineated for the 2 cm tumor containing the ionization chamber insert and these were expanded by 0.6 cm isotropically to create a clinical target volume (CTV). Locally established PTV margins were applied depending on the imaging modality: for the 3D images CTVs were expanded by 0.8 cm axially and 1.2 cm longitudinally; for 4DCT‐P and 4DCT‐A images reduced margins were used of 0.6 cm axially and 0.8 cm longitudinally. Therefore, the total expansion GTV–PTV was 1.4 cm/1.8 cm (3DCT) and 1.2 cm/1.4 cm (4DCT). (Note that differential PTV margins for 3D and 4D cases are well‐established practice, consistent with several current lung radiotherapy trial protocols^21^, ^22^, ^23^ and other relevant guidance.^24^) After all expansions had been applied, the mean PTV volumes used in this study were 67.4 ± 6.1 cm^3^ (3DCT), 65.6 ± 7.0 cm^3^ (4DCT‐A), and 68.2 ± 6.2 cm^3^ (4DCT‐P). VMAT plans were then created for a Varian TrueBeam linac featuring Millennium 120 MLCs with dynamic jaw tracking. The beam energy used was 6 MV, with a nominal dose rate of 600 MU/min and all plans were calculated using the Eclipse AcurosXB dose calculation algorithm (v13.6.23). Plans were optimized in Eclipse against each PTV/image pair as per local protocol; (1) laterally offset isocenter, (2) full rotations with optimized collimator settings, and basic PTV coverage and normal tissue sparing and MU objectives (no organs at risk were outlined for this phantom). For the 4D images, all doses were calculated on the AIP. In each case, a total dose of 55 Gy in 20 fractions was prescribed (2.75 Gy/#), and the expected dose at the center of the GTV determined as calculated by the TPS.

#### 2.3.2 Dosimetry

Finally, each treatment plan was delivered on a Varian TrueBeam linac to the moving phantom, into which was inserted a PTW 0.015 cm^3^ PinPoint ionization chamber. The chamber was calibrated by performing test exposures of the phantom without movement, using static calibration fields that could be compared back to the TPS. In the TPS, the expected dose was derived for all deliveries by taking the average dose over a small volume ion chamber (VOI) delineated to contain the chamber sensitive volume. During delivery of each plan, the relevant breathing trace used at scanning was replayed. As at scanning, no specific section of the breathing trace was targeted for “beam on”, and three fractions of each treatment plan were delivered to mitigate against any interplay effects that may confound a single measurement.^8^, ^25^, ^26^, ^27^, ^28^, ^29^ Therefore in total over 100 treatment deliveries were completed, not including calibration fields. To this end, treatment was delivered effectively randomly over multiple portions of each breathing trace. For each delivery, a charge reading was obtained from the ion chamber, and this was calibrated to dose via a static calibration field delivered to the static phantom and calculated in the TPS.

## 3 RESULTS

### 3.1 Tumor motion representation

**FIGURE 4:**
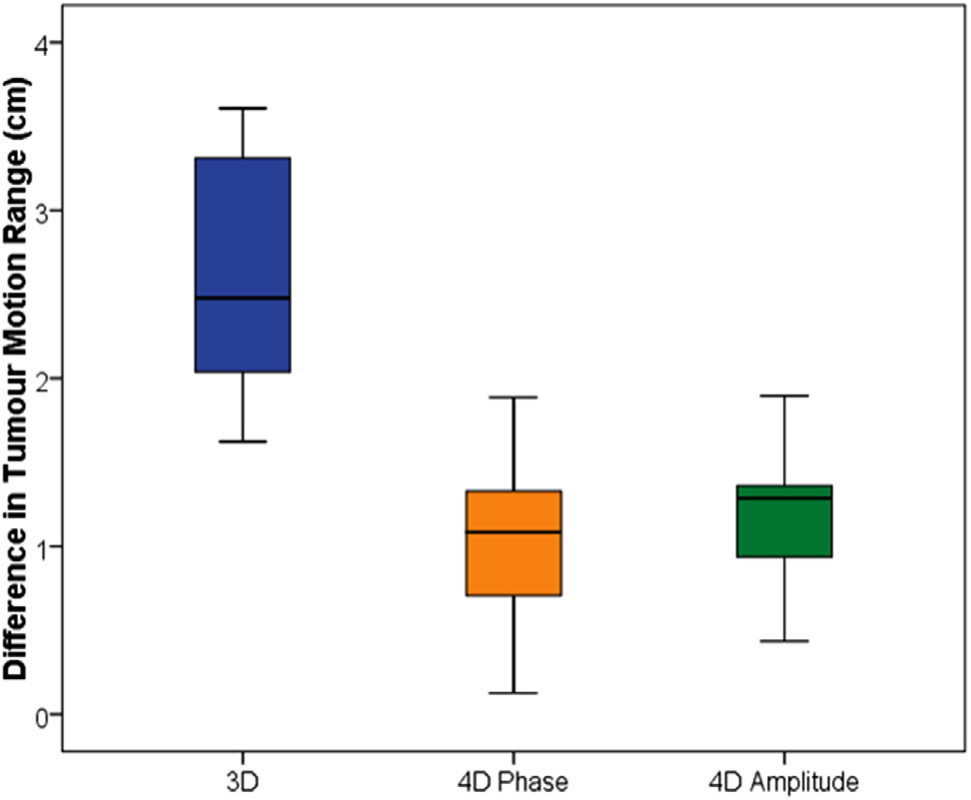
Box plot showing difference between expected and recorded tumor motion range 3DCT (blue), 4DCT‐P (orange), and 4DCT‐A (green) (0 cm being ideal)

Figure 4 shows the difference between expected and measured tumor motion range (expected–measured). For the irregular breathing traces, the median difference in the tumor motion range for 3DCT, 4DCT‐P, and 4DCT‐A was 2.5 [1.6–3.6] cm, 1.1 [0.1–1.9] cm, and 1.3 [0.4–1.9] cm, respectively. This compares with an error of ±0.15 cm on 4DCT‐P for the “SINE” breathing trace, which is thought to be due predominantly to the thresholding technique used for delineation. Therefore even in the case of irregular breathing, the 4D scans better represented the tumor motion compared to 3DCT (*p* = 0.005, 4DCT‐P vs. 3DCT). There was no significant difference detected between phase and amplitude binning (*p* = 0.050).

### 3.2 Time‐averaged density representation

**FIGURE 5:**
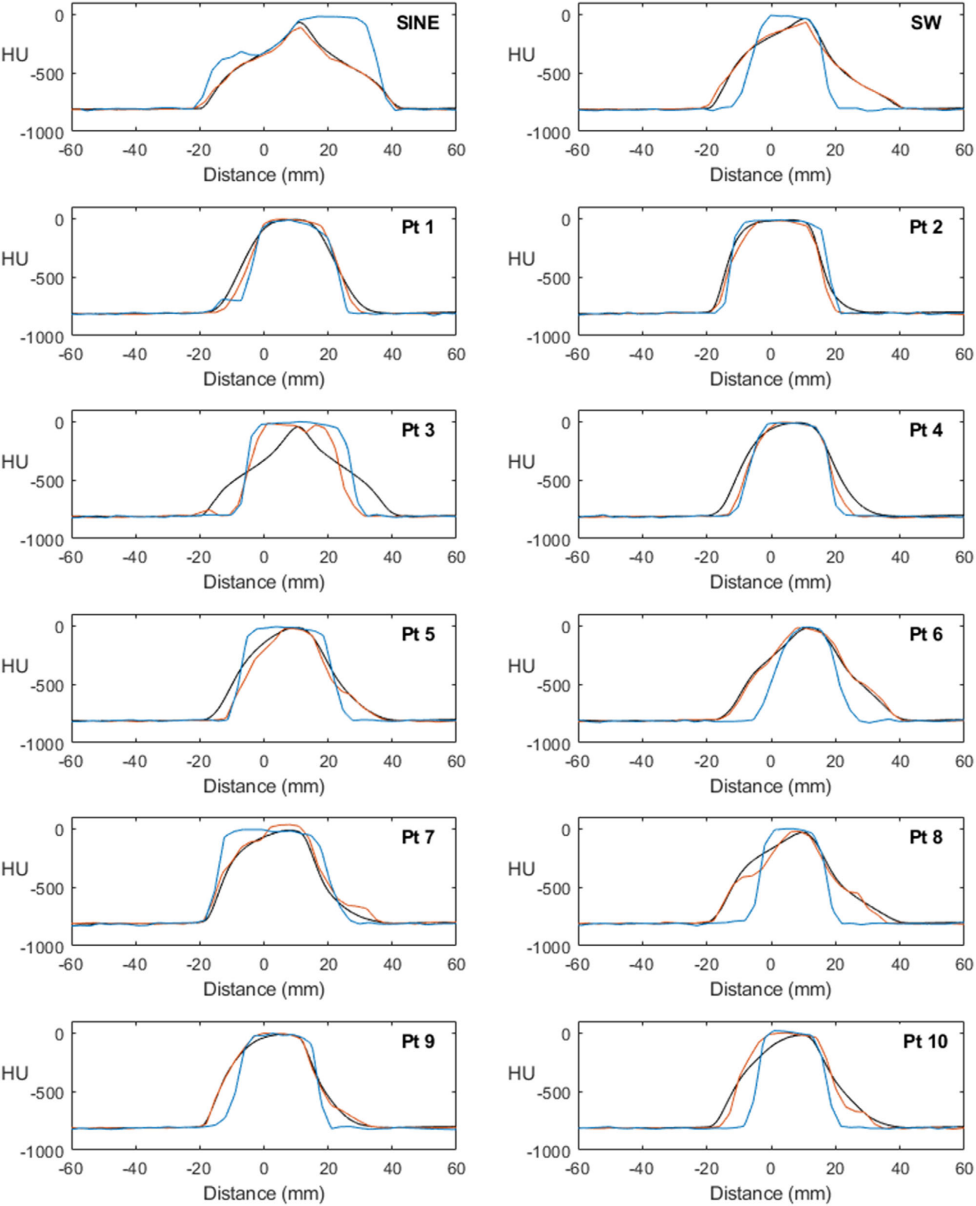
Time‐averaged density profiles for the 10 irregular breathing traces, sine wave, and regular breathing trace, comparing expected profile (black) to measured profiles for 3DCT (blue) and 4DCT‐P (orange)

**FIGURE 6:**
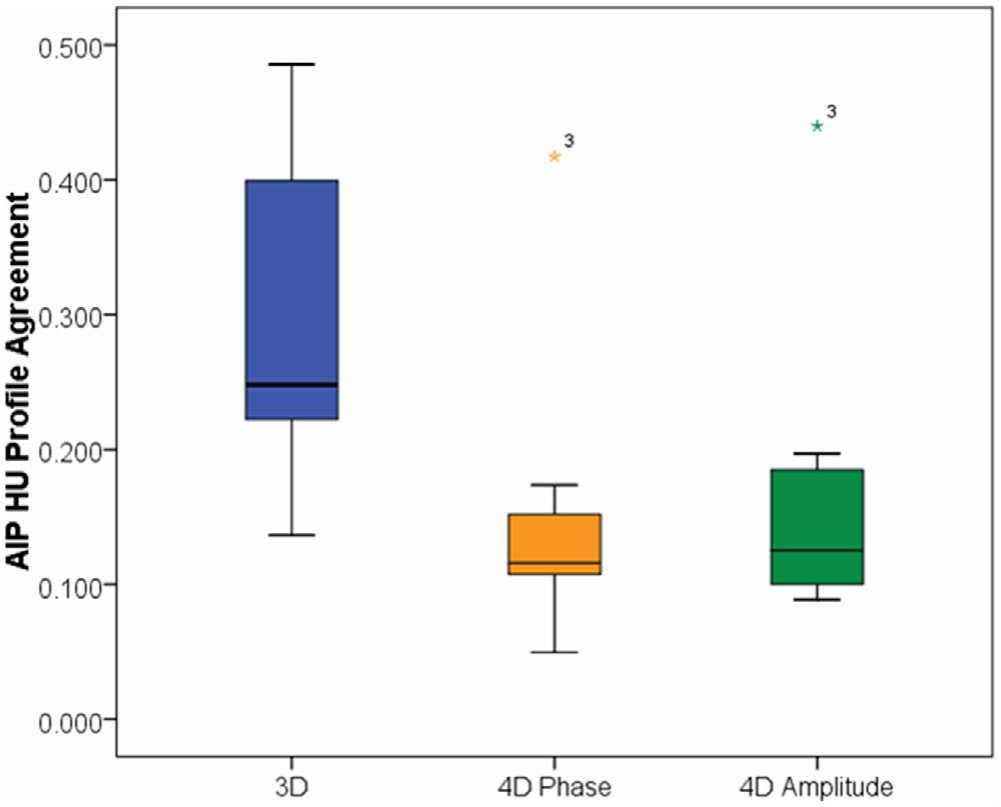
Box plot showing the HU profile agreement assessed via AUC for 3DCT (blue), 4DCT‐P AIP (orange), and 4DCT‐A AIP (green)

The measured and expected density profiles for the 10 irregular breathing traces, sine wave and single regular breathing trace are shown in Figure 5 for 3DCT (blue) and 4DCT‐P (orange) and compared to the expected density profile (black). 4DCT‐A was qualitatively very similar to 4DCT‐P and is not shown for simplicity. Clearly, the 3D image typically incorrectly represents tumor density and position due to being a snapshot of the displacement, whereas the 4D images more accurately represent the time‐averaged densities, even in the presence of irregular breathing. Notably, breathing trace 3 was poorly represented in all images. This patient had a very low respiratory rate of 4 breaths per minute which was slower than the lowest pitch setting available on the Brilliance CT scanner—resulting in gross undersampling of the respiratory motion. Figure 6 shows the AUC scores for each profile comparison for the irregular breathing traces. Median AUC scores (ideal = 0) were 0.25 [0.14–0.49] (3DCT), 0.12 [0.05–0.42] (4DCT‐P), and 0.13 [0.09–0.44] (4DCT‐A). Therefore, 4D scans better represented the average density of the tumor (*p* = 0.005, 4DCT‐P vs. 3DCT). There was no significant difference detected between phase and amplitude binning (*p* = 0.508).

### 3.3 Dosimetric accuracy

**FIGURE 7:**
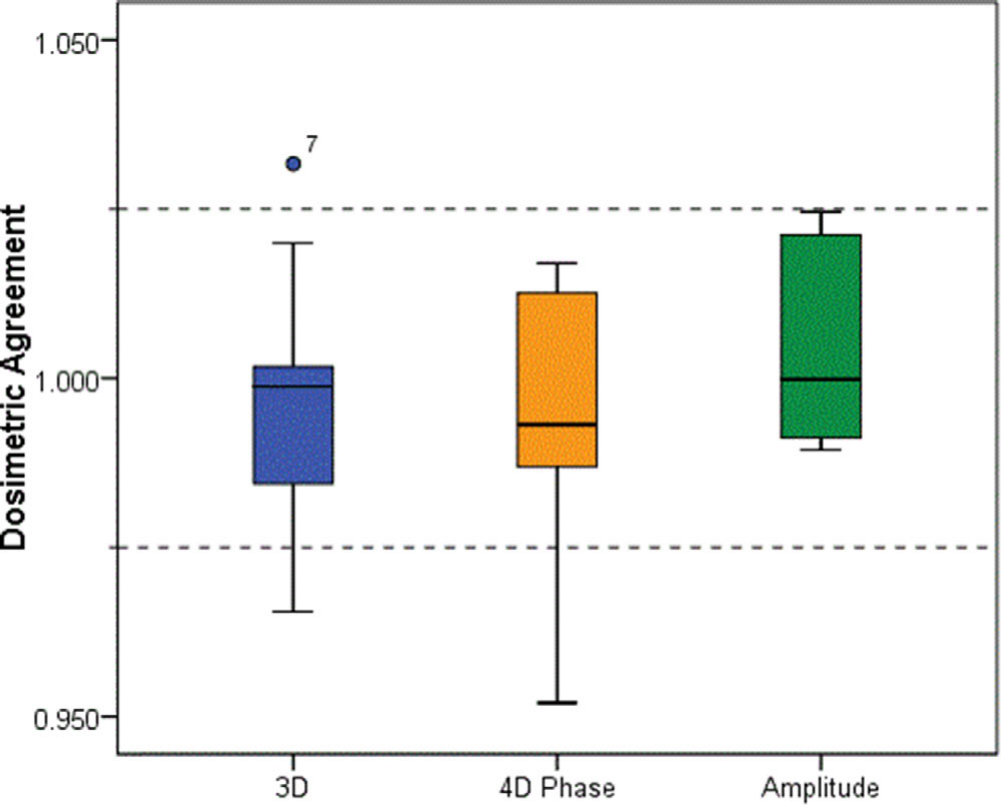
Box plot for VMAT plans, 3DCT (blue), 4DCT‐P (orange), and 4DCT‐A (green), showing dosimetric agreement between TPS and measurement. Dashed lines indicate nominal 2.5% tolerance level

Figure 7 shows the relative agreement between expected and measured doses averaged over three fractions at the center of the GTV/iGTV (expected/measured) for the irregular breathing traces within each imaging modality. Nine of ten 4DCT‐P plans and all 4DCT‐A plans were measured to be within 2.5% of the expected dose, compared with seven of ten 3DCT plans. For reference, the “SINE” trace showed agreement within 2% for both 3DCT and 4DCT images, and the “SW” trace agreed within 1.3% for the 4DCT images but 5% for the 3DCT. This suggests that the disagreement shown in the 4DCT irregular breathing cohorts is comparable with or only marginally greater than the inherent measurement uncertainty under ideal conditions. The outlier in the 4DCT‐P group was again the very slow breathing trace discussed above. Median dosimetric agreement was close to unity in all cases and therefore not found to be significantly different between modalities.

## 4 DISCUSSION

In this study, we evaluated the relative utility of three commonly available clinical imaging modalities (3DCT, 4DCT‐P, and 4DCT‐A) for the treatment planning and delivery of radiotherapy for lung cancer when the patient is unable to breathe regularly. In doing so, we have aimed to replicate a typical clinical pathway as closely as possible through the use of real human irregular breathing traces acquired at our clinic, and in all aspects of target delineation, dose prescription, and treatment optimization. In particular, we have avoided the use of synthetically generated irregular breathing traces. Consistent with the work of others we conclude no imaging modality successfully captured the full range of tumor motion, since even for the time‐resolved images the measured internal GTV length was still on average ∼1.3 cm shorter than an expected value derived from a priori knowledge of the tumor volume and motion. Richter et al.^30^ similarly concluded that in the presence of irregular breathing 4DCT represents mean tumor motion instead of maximum tumor amplitude. This is in contrast with the regular sine wave “control” trace in which our outlining technique returned a value for tumor motion that was within 2 mm of the expected amplitude.

The question of whether it is clinically necessary to represent the entire tumor motion envelope via an internal target volume is of at least some debate. Many workers have adopted a different approach, choosing instead to identify the phase of breathing that most closely approximates the time‐averaged tumor position (the “mid‐ventilation” technique), and subsequently using this 3D image for tumor delineation and treatment planning.^31^ Such an approach yields systematically smaller target volumes than the internal volume approach taken here, with correspondingly—and appealingly—smaller irradiated volumes, yet existing outcome data (albeit within the context of stereotactic radiotherapy) suggest the two approaches are clinically equivalent in terms of tumor control.^32^, ^33^ Locally, we supplement the use of 4DCT in pre‐treatment with 4D cone‐beam CT (4D‐CbCT) at the linac,^34^, ^35^ which allows offline verification after #1 that the GTV motion demonstrated during treatment is enclosed by the iGTV surface, or at least the PTV surface. Previous works have shown that tumor movement varies significantly both intra‐ and inter‐fractionally.^15^, ^36^ Since 4D‐CbCT image slices are acquired simultaneously rather than sequentially (as for 4DCT), the relationship between irregularity in breathing trace and the appearance of artefact is fundamentally different. Such an approach therefore provides an independent verification of the fidelity of the pre‐treatment images, and assurance that the tumor receives a therapeutic dose during treatment delivery. Since the conclusion of this study we implemented 4DCT for patients with irregular breathing^37^; a subsequent local audit of corresponding 4D‐CbCT data for these patients indicated PTV contours are sufficiently large and correctly located within the treatment room.

For the purposes of the dose calculation, we developed a novel technique to estimate an expected time‐averaged density profile through the tumor by convolving a static profile with a probability density function derived from the irregular breathing trace. With the exception of one very slow breathing trace, we conclude from our data that even in the presence of irregular breathing the time‐averaged density profile represented in the 4D AIP images provides good agreement with this model‐based prediction, and more so than 3DCT. That there was no appreciable difference between amplitude and phase‐based 4D resorting is of note since only phase‐based resorting would be expected in principle to return a time‐averaged density profile in this way. Such agreement is encouraging for the purposes of accurate dose calculation in the TPS. These data also support the use and resilience of an AIP image for dose calculation, for artifacts related to irregular breathing may appear extreme in one or more of the individual bins, but become quite effectively suppressed when averaging over a large number of bins (in the present case 6 bins, though 10 is more typical clinically).

Despite the high degree of irregularity in our breathing traces, no image gaps were identified in our 4DCT‐A images of the sort described by Abdelnour et al.^38^ Two factors may explain this: First, on‐line review of the bin distribution was performed for every scan to ensure appropriate coverage of the breathing traces. Second, we note that we used a different CT scanner and reconstruction protocol than the one used by those authors. As we were unable to demonstrate any significant difference between 4DCT‐A and 4DCT‐P resorting methods, we have retained the use of 4DCT‐P in our local protocols.

The data obtained from the ionization chamber placed inside the irregularly moving phantom indicate acceptable dosimetric performance of the TPS at the measurement point, since the average disagreement after three fractions was only slightly greater than the measurement uncertainty, and within the tolerance we would apply for other kinds of patient QA, with nearly all measurements within 2.5% for the 4DCT images (compared with seven of ten for 3DCT). Again the very slow breathing trace is highlighted; we hypothesize the gross under‐sampling introduced at 4D scanning by using a pitch that was too high (the lowest available pitch on our scanner) resulted in an irradiated volume that was evidently too small, and subsequently an appreciable portion of the tumor trajectory was spent in the low dose region. Although problematic from a clinical implementation perspective, the negative result provides assurance that our measurement technique was at least sensitive to errors of this type.

With respect to the dosimetric results, it is legitimate to ask: to what extent do the PTV margins used compensate for the measured motion deficit? The margins in this study reflect those established locally based on an internal audit of uncertainties and on‐treatment set‐up corrections, including uncertainty in respiration. The total longitudinal expansion for 3DCT (2 × 1.8 cm, at sup and inf ends combined) exceeds 4DCT (2 × 1.4 cm) as it is assumed that the ICRU “internal margin” is already incorporated into the 4D iGTV. So, in both 3D and 4D cases the total PTV extension (3.6 cm 3DCT, 2.8 cm 4DCT) exceeded the measured motion deficit (2.5 cm 3DCT, 1.1 and 1.3 cm 4DCT). Therefore, we suggest that this does not (of itself) account for any dosimetric differences observed. Moreover, PTV volumes were also quite similar across the three modalities (67.4 ± 6.1 cm^3^ (3DCT), 65.6 ± 7.0 cm^3^ (4DCT‐A), and 68.2 ± 6.2 cm^3^ (4DCT‐P)). Perhaps of more relevance is the possibility in the 3D case of capturing the tumor at one end of its motion envelope, as compared with 4D, which renders a truer “time‐averaged” position. In our study of time‐averaged density representation, we saw that 4D performed better in this respect even for the irregular motions. Therefore, the 3D margins, even though generous, might be applied incorrectly. Either way, we saw only weak differences in dosimetric performance between 4DCT and 3DCT concluding that the median agreement was not significantly different between modalities. In our opinion therefore, the value of our dosimetric result is not so much in demonstrating dosimetric superiority for 4DCT, but instead showing that with standard “regular breather” PTV margins 4DCT remain dosimetrically feasible, even in the presence of irregular breathing traces. In particular, this avoids the need for patient‐individualized PTV margins, which are difficult to implement in practice. Further, and in so much as this represents an opportunity to standardize clinical practice across our cohort of lung patients, this can help reduce the risk of a clinical error.^39^, ^40^

There are few studies that have performed similar dosimetric investigations for irregular breathers which limit our ability to put these data in context. Mutaf et al.^13^ conducted a retrospective study of 23 lung cancer patients in which irregular breathing motion was simulated within a TPS. These authors concluded that for “characteristic” irregular motion—in which the irregularities during 4DCT are reproduced at treatment—the dosimetric effects on target coverage (*D*_min_) were minimal at ∼2.5%—so therefore largely consistent with our findings. However, they also advise caution in the case of “uncharacteristic” irregularities—where there is a *systematic* shift in CTV motion between imaging and treatment. In this case, a much larger drop in CTV coverage was reported, on the order of 10%. Richter et al.^30^ carried out a detailed study of the accumulated dose in a tumor surrogate moving both regularly and irregularly, using both ionization chamber and radiochromic film. Conversely, these authors showed that while the tumor movement significantly affects the peripheral target dose relative to the center, this can be adequately modeled and ultimately compensated with the adoption of appropriate PTV margins. Santhanam et al.^41^ carried out a similar phantom study showing a mean disagreement of 2% in the high‐dose region of a programmable moving phantom, and acceptably elsewhere. Finally, Pan et al.^42^ investigated the irregular breathing effect on target doses within a moving phantom in the lung SBRT context. These authors concluded that the dosimetric agreement as assessed—via gamma analysis of radiochromic film—was acceptable for VMAT under gated treatment delivery only. However, these data were acquired after only one fraction of treatment, under which interplay effects can have a more significant effect (and arguably are more relevant within a hypofractionated SBRT context).^8^, ^25^, ^26^, ^27^, ^28^, ^29^

Since our irregular breathing traces were “re‐played” for both imaging and treatment, our study arguably only addresses the first category of characteristic irregularity identified by Mutaf et al.^13^ Moreover, in our phantom we verified dose at the tumor center, so cannot fully address the issue of target coverage and peripheral dose. To the first point, baseline drifts were seen in some of our breathing traces; so when coupled with the random time points chosen for both imaging and treatment, together with the delivery of multiple fractions, this at least supports the notion that some systematic errors were propagated through to treatment delivery. An obvious extension to our study would be to deliberately play back a different breathing trace at treatment than was used for scanning, and then further to supplement the chamber measurements we have made with 2D dosimetry situated in a plane parallel with the direction of motion. Such considerations further highlight the utility of 4D imaging at treatment for the direct comparison of tumor trajectories back to the apparent trajectory in the planning scan, affording an important opportunity for corrective action if required.

The tumor motion ranges used in this study (2.85 cm total platform travel, or 2.1 ± 0.36 cm mean amplitude excluding outliers) might be considered to be at the larger end of typical motion trajectories encountered clinically. Locally, characteristic clinical amplitudes are found to vary from minimal (e.g., for upper lobe/apical tumors, or those tethered to a chest wall or mediastinal structure) up to about 3 cm for more mobile lower lobe tumors that are close to diaphragm. Some particularly mobile tumors may be subject to motion mitigation strategies such as gating or abdominal compression, although equally not all patients can tolerate or comply with such measures. It is nonetheless relevant to ask whether for more moderate motion envelopes of 1–1.5 cm any differences seen here between 4DCT and 3DCT would be as pronounced. Although this was not explored in our study, we suspect that any differences would be less pronounced at smaller amplitudes: for example, in the completely static case there should be no difference between the modalities with the respect to the outcomes we have measured. We therefore take the view that, by showing that 4DCT remains feasible in a relatively highly mobile scenario, clinicians may have confidence in its use across a range of clinical cases, should they choose.

There are several limitations to our study. First, our phantom moved only in one direction (longitudinally), and tumor displacement was rigid rather than deformable. This differs from the clinical scenario, where the tumor movement occurs deformably within the thorax, and in general with components orthogonal to the scanning direction. These differences may have a bearing on the quality and dosimetric performance of the acquired images when breathing is irregular. Further, we have assumed that the external surrogate used for image reconstruction is well correlated with the phantom motion assumed in the analysis, introducing an additional uncertainty in our findings. Second, this study is based on one scanner type and protocol, and one treatment planning and delivery platform. Different acquisition settings, treatment techniques, and dose calculation algorithms may have influenced our results. Finally, we have not considered in this work any formal definition of breathing irregularity or attempted to systematically classify irregularity by phase or amplitude; we have instead found that for all our breathing traces dosimetric discrepancies were acceptable or easily explained. A further study to retrospectively classify our data by the various types of irregular breathing could be proposed. However, we have no facility in our clinic to perform such analyses prospectively, limiting the operational usefulness of such a classification.

## 5 CONCLUSION

For the irregular breathing traces studied tumor motion range and average density was better represented with 4DCT compared with 3DCT. 4DCT images allowed for delivery of VMAT plans with acceptable dosimetric accuracy. No significant differences were detected between phase and amplitude binning. In combination with 4D cone‐beam imaging for treatment verification, our findings gave us confidence to introduce 4DCT and VMAT planning for lung radiotherapy patients with irregular breathing, affording an opportunity to standardize clinical practice across our lung cohort to this group of patients. Caution is advised when breathing traces are very slow and below the recommended pitch settings on the scanner.

## CONFLICT OF INTEREST

The authors declare that there is no conflict of interest that could be perceived as prejudicing the impartiality of the research reported.

## AUTHOR CONTRIBUTIONS

Rhydian Caines contributed to study design, data collection, analysis and interpretation, and drafted the manuscript. Naomi Sisson contributed to study design, data collection, analysis and interpretation, and critical revision of the manuscript. Carl Rowbottom contributed to study design, study supervision and data interpretation, and critical revision of the manuscript. All authors discussed the results and gave final approval of the submitted article.

## Data Availability

The data that support the findings of this study are available from the corresponding author upon reasonable request.
